# Manual segmentation of opacities and consolidations on CT of long COVID patients from multiple annotators

**DOI:** 10.1038/s41597-025-04709-2

**Published:** 2025-03-07

**Authors:** Diedre S. Carmo, Alejandro A. Pezzulo, Raul A. Villacreses, McKenna L. Eisenbeisz, Rachel L. Anderson, Sarah E. Van Dorin, Letícia Rittner, Roberto A. Lotufo, Sarah E. Gerard, Joseph M. Reinhardt, Alejandro P. Comellas

**Affiliations:** 1 School of Electrical and Computer Engineering, Universidade Estadual de Campinas, Campinas, 13083-852 Brazil; 2 Department of Internal Medicine, University of Iowa, Iowa City, 52242 USA; 3 Roy J. Carver Department of Biomedical Engineering, University of Iowa, Iowa City, 52242 USA

**Keywords:** Biomedical engineering, Scientific data

## Abstract

The field of supervised automated medical imaging segmentation suffers from relatively small datasets with ground truth labels. This is especially true for challenging segmentation problems that target structures with low contrast and ambiguous boundaries, such as ground glass opacities and consolidation in chest computed tomography images. In this work, we make available the first public dataset of ground glass opacity and consolidation in the lungs of Long COVID patients. The Long COVID Iowa-UNICAMP dataset (LongCIU) was built by three independent expert annotators, blindly segmenting the same 90 selected axial slices manually, without using any automated initialization. The public dataset includes the final consensus segmentation in addition to the individual segmentation from each annotator (360 slices total). This dataset is a valuable resource for training and validating new automated segmentation methods and for studying interrater uncertainty in the segmentation of lung opacities in computed tomography.

## Background & Summary

Deep learning has revolutionized the field of medical image analysis across various organs and imaging modalities^1^. Recent methods developed for high-resolution chest computed tomography (CT) analysis can automate tasks such as lung parenchyma and lung lobe segmentation^2^, fissure detection and integrity quantification^3,4^, lung nodule detection and segmentation, pneumonia classification, outcome prediction^5^, opacity segmentation, and others^6^. Moreover, recent research has demonstrated that individuals with a prior COVID-19 diagnosis can have lung opacities present for months after the acute infection^7^. In this context, the development of automated methods for the analysis of ground glass opacities (GGO) and consolidations in the lung is paramount to analyze the severity of disease and quantifying lung involvement^8^. These methods pose a promising future for automated decision support in medical pipelines and automated analysis of large research cohorts, accelerating medical research and patient care.

Supervised deep learning methods for automated image segmentation rely on manual annotations, which are considered ground truth for development and validating new algorithms. Manual segmentation of structures and findings in high-resolution CT is tedious, time consuming, and subject to human error. Furthermore, some structures are more challenging to segment due to low contrast and/or ambiguous boundaries^9^. For example, the lung parenchyma boundary is well defined by the Hounsfield unit difference between soft tissue and the air filled lung in most cases, with lung ground truth being readily available from many sources in the literature or reliably obtained through automated methods^10^. In contrast, segmentation of opacities in severe cases of COVID-19 pneumonia, is challenging because the opacities can be localized in any part of the lung and the boundaries are often ambiguous^11^. The task of discriminating opacities into consolidation and GGO subtypes is even more challenging due to the additional uncertain boundaries between adjacent GGO and consolidated regions^12^.

Existing public datasets provide manual annotations of lung opacities in CT images^13–15^. However, manual annotations of multilabel opacities, i.e., distinguishing GGO vs. consolidation with separate labels, is not readily available in public datasets. The task of GGO vs. consolidation segmentation is challenging due to to ambiguous borders between the two regions. Furthermore, distinguishing consolidation and blood vessels is difficult on CT. Currently, there are only two public datasets containing manual annotations of GGO and consolidations separately, which we have labeled Medical Segmentation COVID (MSC), following the original source^16^ and SemiSeg, following the name and data organization proposed by Fan *et al*.^12^. These datasets have been used in a large number of research studies for the optimization of supervised and semi-supervised deep learning methods for automated GGO and consolidation segmentation^12,17–22^. Both datasets included chest CT scans of acute COVID-19 patients and accompanying annotations. MSC annotated 373 slices from public scans made available by The Italian Society of Medical and Interventional Radiology and SemiSeg annotated 100 slices originating from RadioPaedia public images, with annotations performed by a single expert according to their website^23^. Given that those datasets used scans from acute COVID-19 patients, there is no dataset providing this type of annotation in CT scans of long COVID patients, i.e., images of subjects with persistent symptoms even months after the acute infection has subsided.

**Fig. 1:**
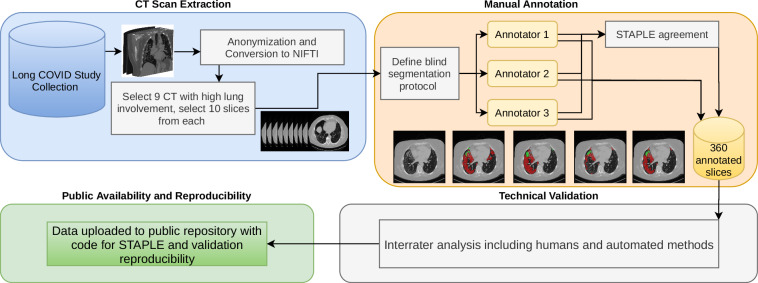
Overview of the process of creating this dataset, from selecting the slices to be segmented, through blind multiple rater segmentation and consensus, technical validation and making the data and involved code public.

In this work, we built the Long COVID Iowa-UNICAMP dataset (LongCIU): a novel dataset consisting of 90 axial CT slices with multiple expert annotations of GGO and consolidations in the lung. Every image has the presence of at least GGO. Each image was annotated blindly by three groups of two annotators each. In each group, the MD with less experience worked on the fully manual annotation, and the pulmonologist MD with more experience verified the result. All three groups worked on the same axial slices, without using any automated method as reference or initialization. A final consensus annotation was created by applying the STAPLE algorithm^24^ and the individual annotations from each of the three expert annotators are included in the public dataset, resulting in a total of 360 slices (Fig. 1). This allows future research not only to compute metrics against the consensus annotation but also to compute their interrater agreement in comparison with manual annotation. The LongCIU dataset will promote a deeper understanding of human uncertainty in GGO and consolidation segmentation in CT scans.

## Methods

### Acquisition

CT scan data was originally collected at the University of Iowa Hospitals and Clinics^7^, in an ongoing Long COVID study. Adults with history of COVID-19 infection confirmed by a positive antigen or reverse transcriptase-polymerase chain reaction that remained symptomatic 30 days or more following diagnosis of acute infection, were prospectively enrolled starting in June 2020. Chest CT scans were conducted with Siemens scanners. All scans employed tube current modulation. A standardized protocol for noncontrast chest CT imaging was followed, which entailed acquiring an inspiratory scan at total lung capacity (TLC) and an expiratory scan at residual volume (RV). Reconstruction of images was performed using iterative reconstruction techniques with a section thickness × interval of 1 × 0.5 mm. This study was performed in line with the principles of the Declaration of Helsinki. Study protocols were approved by the University of Iowa institutional review board (IRB), case number 202005421, and were Health Insurance Portability and Protection Act-compliant. Participants were required to sign written informed consent before inclusion, including the possibility of sharing anonymized images. The construction of the LongCIU dataset is a retrospective, anonymous use of part of this data acquisition.

### Manual Annotation and STAPLE Consensus

Since patient information is not necessary for segmentation, images were completely anonymized and converted to NIFTI format. To make the time needed for fully manual segmentation feasible, 9 TLC CT scans were selected from the whole study cohort. For the selection criteria, given that most Long COVID patients do not present lung opacities, an automated method^22^ was used to quantify lung opacity involvement. The top-9 scans regarding involvement were selected as sources for slices to be segmented. Six physicians participated in the annotation process. All physicians participated in a joint training session and agreed to a segmentation protocol. To avoid bias from automated methods and better understand human interrater agreement in this task, we chose to not use any initial automated segmentation as initialization. The manual segmentation would be performed from scratch, following the ground glass opacity and consolidation definitions from Silva *et al*.^25^. Opacities correspond to parenchyma areas of greater density as a result of lung inflammation due to infectious processes. Therefore, opacities present higher Hounsfield unit intensity, and are distinguishable from surrounding tissue and structures. In GGOs, even with increased density of the lung parenchyma, it is still possible to identify the contours of the vessels and bronchi within. In consolidations, the increased attenuation makes the underlying anatomy invisible, usually associated with greater disease severity.

**Fig. 2:**
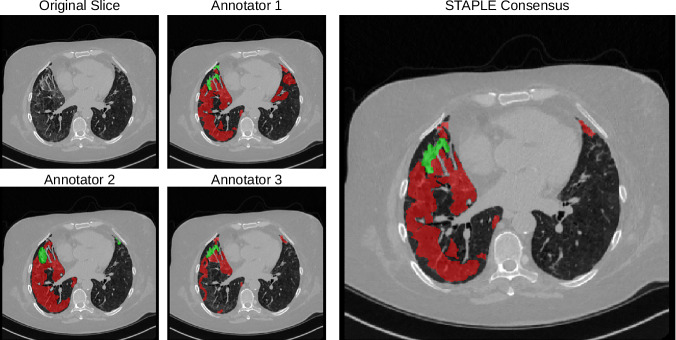
A sample slice and its accompanying segmentations, with GGO in red and consolidation in green. Notice how STAPLE arrives at a final consensus with a opacity sensitivity higher than annotator 3 but lower than annotator 1. The difficulty of differentiating vessels from consolidation is also illustrated in the anterior region of the right lung.

Following these definitions, the annotators agreed to a selection of 10 axial slices from each CT scan. Some slices are adjacent, and some are not. Every slice has the presence of at least GGO. After the joint session, physicians were divided into three pairs. Each pair was composed of one expert board certified pulmonologist with more than 10 years of experience, and one post medical school resident trainee. Each pair worked blindly from the other groups, with the trainees annotating images under the continuous supervision and revision of the expert. Each pair is referred to as annotator 1, 2 and 3 for the remainder of this paper. From this point forward, the annotators segmented all 90 slices blindly from each other, using the 3D Slicer software^26^. A video tutorial was provided to each annotator illustrating the segmentation process and how to use 3D Slicer, including how to set and use Hounsfield unit windows and the segmentation tools. With 90 slices annotated 3 times, we computed the STAPLE consensus per slice for a final segmentation map using the implementation provided by SimpleITK^27^ (Fig. 2). First, STAPLE was computed for the general opacity area (GGO + consolidation). Then, the STAPLE consensus only for consolidation is computed separately. Finally, areas that are considered consolidation and opacity were marked as consolidation (label 2), and the remaining opacity area was marked as GGO (label 1).

## Data Records

**Fig. 3:**
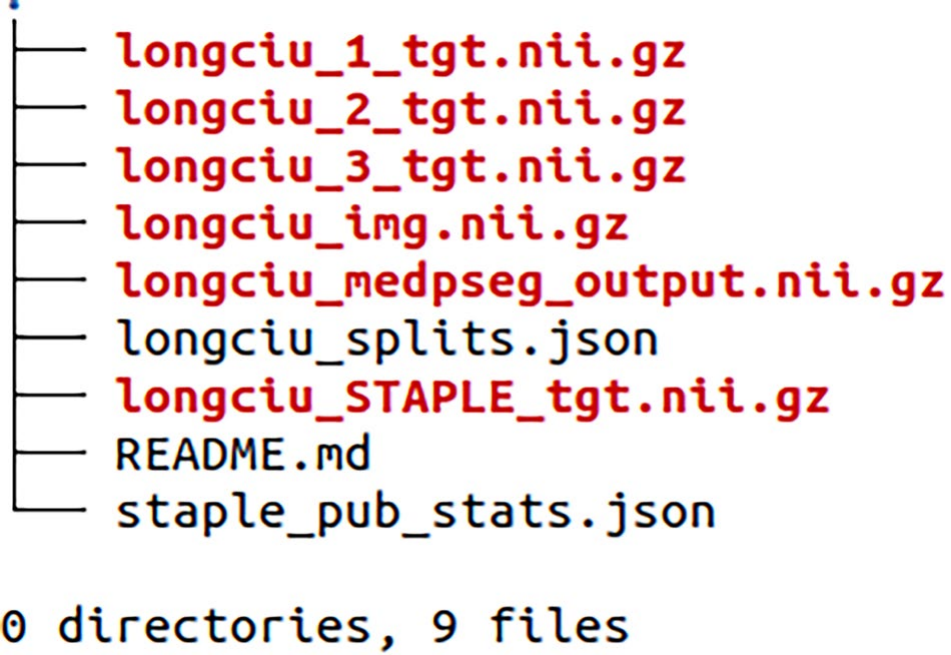
File tree for the dataset, including images, annotations per annotator and consensus, MEDPSeg output, optional proposed training splits, and STAPLE statistics.

The data generated by this research is publicly available in the Iowa Research Online (IRO) repository and can be accessed at the following URL: 10.25820/data.007301^28^. The data folder structure is as follows (Fig. 3). Available files include the 90 anonymized CT slices in original Hounsfield unit intensities, 90 manual segmentation masks from each annotator, and the 90 STAPLE consensus among the three annotators, for a total of 360 segmentation masks. The STAPLE consensus should be considered the final ground truth for the purpose of training or validating automated methods. Integer label 1 corresponds to GGO, and integer label 2 corresponds to consolidation in the provided masks. The accompanying code for reproducing our technical validation and the STAPLE consensus is made available separately in https://github.com/MICLab-Unicamp/LongCIU, including all expected outputs.

Regarding the adjacency of the involved slices and their relative position in the original 3D scan, Table 1 details the original 1-indexed position of axial slices and their corresponding index on the provided 90 slices.

**Table 1:** Each row corresponds to a different subject (ID) as source for 10 slices.

ID	Slices	Axial Slice N
1	1-10	242, 243, 244, 269, 270, 271, 193, 194, 195, 196
2	11-20	224, 225, 226, 227, 228, 341, 342, 343, 344, 345
3	21-30	197, 198, 199, 200, 253, 254, 255, 256, 279, 280
4	31-40	209, 210, 211, 212, 326, 327, 328, 257, 258, 259
5	41-50	230, 231, 232, 233, 254, 255, 256, 284, 285, 286
6	51-60	174, 175, 176, 177, 208, 209, 210, 130, 131, 132
7	61-70	238, 239, 240, 241, 214, 215, 216, 217, 218, 241
8	71-80	213, 214, 215, 235, 236, 237, 196, 197, 198, 199
9	81-90	117, 118, 119, 120, 197, 198, 199, 257, 258, 259

Axial slice index (N) reveals adjacency information.

## Technical Validation

**Fig. 4:**
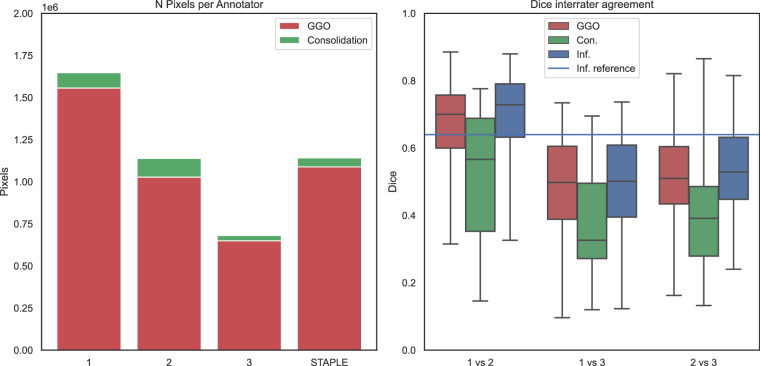
On the left, number of pixels classified as GGO and consolidation for each annotator. On the right, agreement Dice overlap between annotators 2 and 3, 1 and 3, 1 and 2 for overall infection in blue, (Inf = GGO + consolidation), consolidation only in green (Con.) and GGO in red. The horizontal blue line corresponds to the inter-human annotator agreement for overall infection reported by Sotudeh-Paima *et al*.^31^.

**Fig. 5:**
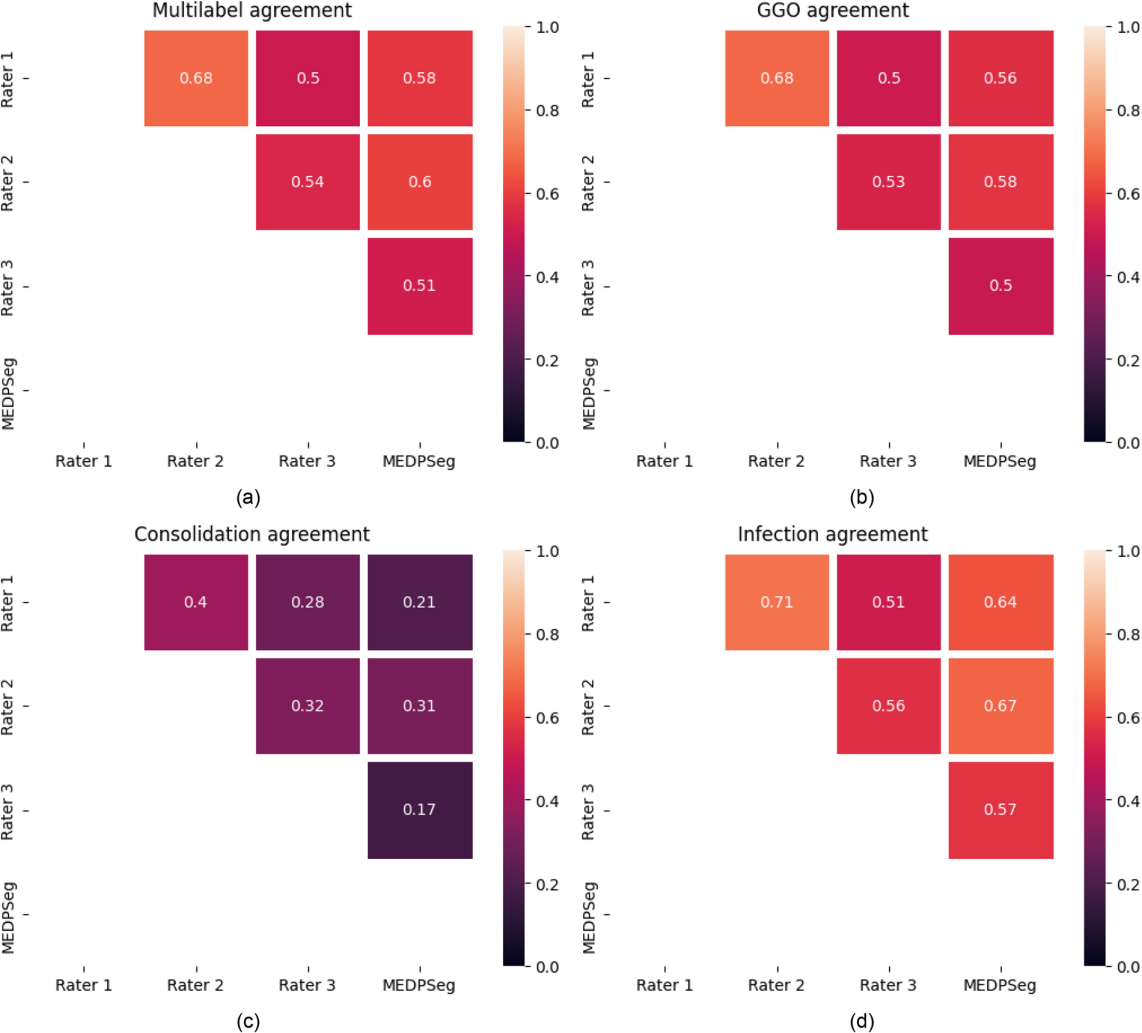
Cohen’s Kappa interrater agreement between the three human raters and the MEDPSeg automated method for (**a**) The multilabel agreement, (**b**) GGO only, (**c**) consolidation only, and (**d**) overall infection, (GGO + Consolidation).

To validate the technical quality of the generated data, we computed number of pixels annotated for each annotator, Dice^29^ agreement among annotators (Fig. 4), and Cohen’s Kappa^30^ interrater agreement, per class, also involving MEDPSeg^22^’s blind performance as an automated method (Fig. 5).

When looking at the number of pixels annotated by each annotator, we notice different sensitivity levels among the annotators. Through the STAPLE consensus and thanks to having three different opinions, we were able to achieve a middle ground among the three expert judgments. When comparing each annotator with each other, we notice overall infection Dice agreement similar to the 64% human agreement reported by Sotudeh-Paima *et al*.^31^. We have found no report from other research about the human interrater agreement when dealing with GGO and consolidation separation.

Due to the low prevalence of consolidations in Long COVID patients, we noticed strong disagreement among annotators in consolidation segmentation. Confusion matrices were constructed to visualize the Cohen’s kappa score interrater agreement among annotators for each class. We computed Cohen’s kappa in four ways: considering each pixel as a multilabel rater decision; looking only at GGO; only at consolidation; and finally looking at the overall opacity or infection agreement. The MEDPSeg automated method which had not seen this dataset during training was also included as a rater. For reference, MEDPSeg achieved Dice coefficients of: 0.64 for overall opacity; 0.57 for GGO; and 0.42 for consolidation, when compared to the STAPLE consensus.

With the reported interrater agreement being close to that reported by the literature in other datasets for overall opacity segmentation, we believe LongCIU is of sufficient technical quality for use by the community. In addition, the performance of an automated method trained in separate data was indistinguishable from humans in the interrater agreement through Cohen’s kappa score. On the other hand, there are strong disagreements among raters in the delineation of consolidations in Long COVID patients. These technical validation results suggest the performance of automated methods are already at the frontier of what is feasible, when taking into consideration human uncertainty in manual annotation of x-ray-based computed tomography. We invite others to further investigate this hypothesis and hope that this data will be useful for related research.

## Usage Notes

The results from each annotator are included for the purposes of uncertainty and interrater analysis. The repository also includes a simple sample implementation of an UNet for supervised training using this dataset, with a suggested data split. Note, however, that we strongly recommend against using only this dataset for training, and encourage the use of this data either as an external testing dataset or as part of semi-supervised approaches. Users are free to concatenate GGO and consolidation into a single, more reliable general infection segmentation if desired. Useful visualization software includes ITK-Snap^32^ or 3D Slicer^26^. The NiBabel^33^ or SimpleITK^27^ libraries can be used to read .nii.gz files programmatically.

## Data Availability

The code for reproducing the STAPLE consensus and all technical validation figures in this paper is available at https://github.com/MICLab-Unicamp/LongCIU. Used parameters and output STAPLE statistics are in the repository for reproducibility.
